# Imprinted Contact Lenses for Ocular Administration of Antiviral Drugs

**DOI:** 10.3390/polym12092026

**Published:** 2020-09-04

**Authors:** Angela Varela-Garcia, José Luis Gomez-Amoza, Angel Concheiro, Carmen Alvarez-Lorenzo

**Affiliations:** Departamento de Farmacología, Farmacia y Tecnología Farmacéutica, I+D Farma Group, Facultad de Farmacia and Health Research Institute of Santiago de Compostela (IDIS), Universidade de Santiago de Compostela, 15782 Santiago de Compostela, Spain; angela.varela.garcia@rai.usc.es (A.V.-G.); joseluis.gomez.amoza@usc.es (J.L.G.-A.); angel.concheiro@usc.es (A.C.)

**Keywords:** drug-eluting contact lens, molecularly imprinted hydrogel, antiviral drug, sustained release, cornea penetration, sclera penetration

## Abstract

A variety of ocular diseases are caused by viruses, and most treatments rely on the use of systemic formulations and eye drops. The efficient ocular barriers that oppose antiviral drug penetration have prompted the development of improved topical delivery platforms. The aim was to design hydrogel contact lenses endowed with an affinity for acyclovir (ACV) and its prodrug valacyclovir (VACV), first-choice drugs against herpes simplex virus (HSV) ocular keratitis, and that can sustain the release of therapeutic doses during daily wearing. Functional monomers suitable for interaction with these drugs were screened using computational modeling. Imprinted and non-imprinted hydrogels were prepared with various contents in the functional monomer methacrylic acid (MAA) and characterized in terms of swelling, transmittance, mechanical properties, and ocular compatibility (hen’s egg test on chorioallantoic membrane (HET-CAM) assay). The values were in the range typical of soft contact lenses. Compared to ACV, the capability to load VACV was remarkably higher due to stronger electrostatic interactions with MAA. The advantages of the imprinting technology were evidenced for VACV. Stability of VACV loading solution/hydrogels under steam heat sterilization and subsequent drug release was investigated. Permeability studies through bovine and porcine cornea and sclera of the drug released from the hydrogels revealed that VACV accumulates in the cornea and can easily cross the sclera, which may facilitate the treatment of both anterior and posterior eye segments diseases.

## 1. Introduction

Infection by herpes simplex virus (HSV) starts when the virus comes into contact with damaged skin or mucous membranes; the incubation period extends to 4 days [1]. There are two subtypes of herpes simplex virus, HSV-1 and HSV-2. The main difference is that HSV-1 appears mainly in the orolabial area, while HSV-2 affects the genital area, although, in developed countries, cases of genital conditions due to HSV-1 and orolabial due to HSV-2 are on the increase [2]. It is estimated that 90% of the world’s population is infected with HSV [2]. Periodically, the virus can reactivate and travel to the skin or mucous membranes, causing a recurrent symptomatic or asymptomatic infection. Many factors can trigger this reactivation, for example, stress, exposure to heat or cold, menstruation, fever, or immunosuppression [3]. The clinical manifestations depend on whether the infection is primary or recurrent, the immune status of the host, and the entry portal [4].

At the ocular level, recurrent HSV represents a serious epidemiological cause of infectious and inflammatory disease [5,6]. Herpes disease affects more than 10 million people, and of these, approximately 2 million suffer vision problems in the affected eye. Epithelial keratitis accounts for 50–80% of ocular herpes. Worldwide, about 1 million new or recurrent cases of epithelial keratitis occur annually [7], and it is the most common cause of irreversible blindness in developed countries [8]. Ocular herpes is related to primary orofacial herpes (HSV-1); about 56%–58% of patients with ocular herpes have a history of oral herpes [8,9].

Current therapy for the treatment of HSV ocular keratitis includes topical, oral, and intravenous antiviral agents [3,10]. Viral keratitis can become a chronic and recurrent disease, affecting patients’ quality of life due to the limited efficacy of available treatments [10,11]. Most of the approved antivirals are acyclic nucleosides and nucleotide analogs, which interrupt virus replication [12].

Acyclovir (9-(2-hydroxyethoxymethyl) guanine) (ACV) is a purine nucleoside analog that remains the treatment of choice for HSV-1 infections to date [10,11]. It is a selective antiviral agent as it specifically targets virus-infected cells and selectively inhibits the viral DNA polymerase [4,13]. Nevertheless, the inhibition of virus replication may not affect the latency, so the infection may have not been solved [3,10,12]. ACV has a good safety profile and is well-tolerated by patients, but its oral bioavailability is low (10%–20%) and its plasma half-life is short, which involves frequent administrations [3,4,5]. Moreover, there are studies that demonstrate the growing resistance to this drug mainly in immunosuppressed subjects, developed through a mutation of the viral gene thymidine kinase, essential for the phosphorylation of ACV. An additional limitation of oral administration of ACV is renal toxicity in elderly patients, who are unable to excrete the drug properly [8,10,12]. An alternative is the topical application of ACV, but its effectiveness depends on its ability to cross the epithelium [3]. In comparative studies with other non-selective antiviral agents, ACV ointment has been shown to be more effective and less toxic [13]. The problem with topical forms is their low retention time on the eye surface [10]. In some cases, corticosteroids are used as adjuvant therapy to antivirals [11], but many side effects can occur in long-term therapy, including cataract, suppression of the immune response, and possible secondary glaucoma [10,14].

**Figure 1:**
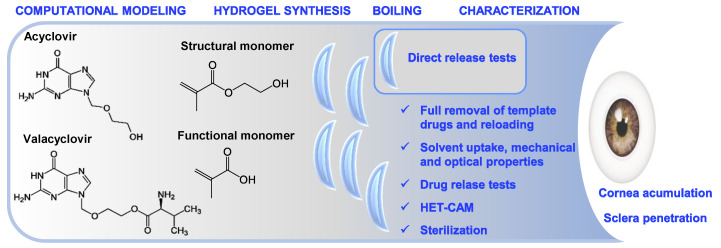
Structure of acyclovir (ACV) and valacyclovir (VACV) and workflow of the experiments.

Valacyclovir (VACV) is a l-valine ester of ACV (Figure 1) and acts as a prodrug with improved bioavailability [13,15,16], but the oral administration of VACV still does not provide effective concentrations in the eye [17]. For example, oral administration of VACV (500 mg/day) does not suppress HSV-1 DNA shedding in tears [16,18]. Reports on topical formulations of VACV are still scarce and, so far, they focused on cationic Eudragit microspheres for mucoadhesion to the cornea surface [15] and solid lipid nanoparticles to penetrate into the eye tissues [19]. Recent studies have confirmed that VACV binds to the oligopeptide transporter of the corneal epithelium, and that its transcorneal permeability is three times higher than that of ACV. It is transformed to ACV by enzymatic hydrolysis in the eye [15,20]. VACV also shows a higher affinity than ACV for the amino acid transporter ATB^0,+^ present in ocular tissues [21].

The aim of this work was to design hydrogels suitable for soft contact lenses (SCLs) with an affinity for ACV and VACV and that can sustainedly release these drugs on the ocular surface during daily wearing (Figure 1). Among the proposed procedures to endow the SCLs with an affinity for specific molecules, the creation of artificial receptors using the molecular imprinting technique stands out [22,23,24]. This technique requires incorporating the drug into the monomers mixture so that the monomers can rearrange according to their affinity. This rearrangement becomes permanent during polymerization. The removal of the template molecules generates cavities with the most appropriate size and chemical groups to host the drug of interest again [25]. The molecular imprinting approach has been successfully applied to develop SCLs loaded with antiglaucoma [22,26], antiallergic [27,28], and antimicrobial [29,30] drugs, among others, and therapeutic agents that may increase ocular comfort [31] and even address the management of diabetic eyes [32,33], but not with antiviral drugs yet. 

To carry out the work, functional monomers suitable for interaction with the antiviral drugs were first screened using computational modeling, a technique that has been shown to be efficient to save time and materials in the development of imprinted materials [34]. Methacrylic acid (MAA) showed a higher affinity for the drugs than the structural monomer 2-hydroxyethyl methacrylate (HEMA) and other functional monomers. MAA may interact with the side chain of VACV through not only hydrogen bonding with the ring (as in the case of ACV) but also electrostatic interactions with the amino group of the valine chain. Hydrogels were prepared with various contents in the functional monomer in the presence (imprinted) and absence (non-imprinted) of each drug. The hydrogels were characterized in terms of swelling, light transmission, mechanical properties, eye compatibility (hen’s egg test on chorioallantoic membrane (HET-CAM) assay), and capability to load and release the antiviral drugs. The stability of VACV loading solution/hydrogels under steam heat sterilization and subsequent drug release was investigated. Finally, the permeability through bovine and porcine cornea and sclera of the drug released from the hydrogels was evaluated (Figure 1).

## 2. Materials and Methods

### 2.1. Materials

Acyclovir (ACV; MW 225.21 g/mol; solubility in water 1.02 mg/mL [35]) was purchased from Farmalabor (Canosa di Puglia, Italy); valacyclovir hydrochloride (VACV; MW 360.80 g/mol; solubility in water 174 mg/mL [36]) was from Acros Organics (Geel, Belgium); 2,2′-azo-bis(isobutyronitrile) (AIBN), dichlorodimethylsilane, ethylene glycol dimethacrylate (EGDMA), and methacrylic acid (MAA) were from Sigma-Aldrich (Steinheim, Germany); ethanol absolute and NaOH were from VWR (Leuven, Belgium); 2-hydroxyethyl methacrylate (HEMA) was from Merck (Darmstadt, Germany); acetic acid and NaCl were from Scharlau (Sentmenat, Spain); and methanol was from Fisher (Loughborough, UK). Ultrapure water (resistivity ˃18 MΩ·cm) was obtained by reverse osmosis (MilliQ^®^, Millipore, Madrid, Spain). Simulated lacrimal fluid (SLF) was prepared with the following composition: 6.78 g/L NaCl, 2.18 g/L NaHCO_3_, 1.38 g/L KCl, and 0.084 g/L CaCl_2_·2H_2_O with pH 7.5. Carbonate buffer pH 7.2 was prepared by mixing buffer solution A (6.2 g/L NaCl, 0.355 g/L KCl, 0.1 g/L NaH_2_PO_4_·H_2_O, and 2.45 g/L NaHCO_3_) and buffer solution B (0.115 g/L CaCl_2_ and 0.155 g/L MgCl_2_·6H_2_O).

### 2.2. Computational Modeling

A preliminary study was carried out using computer modeling to elucidate interactions between the drugs to be studied (ACV and VACV) and functional monomers used in the synthesis of hydrogels. The tested monomers were acrylamide (AAm), 2-aminoethyl methacrylate hydrochloride (AEMA), N-(3-aminopropyl) methacrylamide hydrochloride (APMA), ethylene glycol phenyl ether methacrylate (EGPEM), butoxyethyl methacrylate (BEM), hydroxyethyl methacrylate (HEMA), and methacrylic acid (MAA). The 3D structure of the functional monomers and ACV and VACV was taken from the PubChem database [37]. The SDF files were transformed to PDB files using OpenBabel v. 2.4.1 software [38]. The Autodock Tools v. 4.2.6 software was used to calculate molecular docking. In all cases, the grid was generated with default settings around the monomer and the drug, the smallest conformation was used, and the docking was performed using the Lamarckian Genetic Algorithm [39]. Estimated free energy of binding (*ΔG_binding_*) and dissociation constant (*Ki*) values were obtained. Autodock used a semi-empirical force field to evaluate the binding in two steps. The ligand (e.g., monomer) and receptor (e.g., drug) started in an unbound conformation. First, the intramolecular energetics were estimated for the transition from these unbound states to the conformation in the bound state. Second, the intermolecular energetics of combining the ligand and receptor were estimated. Energies of dispersion/repulsion, hydrogen bonding, electrostatics, and desolvation were evaluated as described in the user guide [40]. The dissociation constant of the complex (*Ki*), also known as the inhibition constant, was estimated as follows:

*Ki* = e^(*ΔGbinding*/(*RT*))^ (1)

In this equation, *R* is the gas constant and *T* the absolute temperature in Kelvin.

### 2.3. Synthesis of Imprinted and Non-Imprinted Hydrogels

Different mixtures of monomers were prepared as shown in Table 1. The components were added to vials and mixed at room temperature and under magnetic agitation (300 rpm) until they were completely dissolved. Finally, the initiator (AIBN) was added, and the solutions were stirred for 15 min more. The solutions were injected, with a needle and syringe, into pre-assembled molds, consisting of two pre-treated glass plates (12 × 14 cm) separated by a 0.45 mm-thick silicone frame. After pre-treatment of the glass plates with dichlorodimethylsilane, the plates were left to dry in a hood for 1 h, thoroughly washed with ethanol, rinsed with water, and dried in an oven at 70 °C for 1 h before being assembled. Polymerization of the monomers inside the molds was carried out for 12 h at 50 °C and then for a further 24 h at 70 °C. All hydrogel compositions were prepared in triplicate.

### 2.4. Drug Removal

After polymerization, each hydrogel sheet was immersed in 500 mL of boiling water for 15 min in order to remove unreacted monomers and template drugs and facilitate the cutting into discs (10 mm in diameter). Further washing was then performed, except for a few discs of each type of hydrogel, which were reserved for the direct drug release test (as explained in Section 2.5). For the washing, the discs were immersed in water, under magnetic agitation (300 rpm) and at room temperature. The medium was replaced every 24 h until no signal was detected in the range of 190–800 nm (UV-Vis spectrophotometer, Agilent 8534, Waldbronn, Germany). When no spectrophotometric signal was detected, the hydrogels were dried in an oven at 70 °C for 24 h and stored protected from light and humidity. In parallel, the amount of ACV (MIP_ACV_, MIP_A1:5_, MIP_A1:10_, and MIP_A1:15_) and VACV (MIP_VACV_, MIP_V1:6_, MIP_V1:12_, and MIP_V1:32_) removed in each washing step was monitored spectrophotometrically at 252 and 253 nm, respectively.

### 2.5. Direct Drug Release Test from Boiled Hydrogels

After the boiling step, three discs of each type of hydrogel were individually placed in vials containing 5 mL of SLF and kept under oscillating agitation (300 rpm) at 35 °C. At preset times (0.5, 1, 2, 6, and 24 h), 3 mL of medium was removed and the absorbance was measured at 252 nm (ACV) and 253 nm (VACV) (UV-Vis spectrophotometer, Agilent 8453, Waldbronn, Germany), returning the samples to the release vial. The experiments were carried out in triplicate. The amounts of drug released were calculated using previously prepared calibration curves and referred to the unit of mass of the dry disc. The calibration curves were prepared by dissolving ACV (30 µg/mL) in ethanol:water (50:50, *v*/*v*) mixture, and VACV (50 µg/mL) in water. Dilutions of 2, 3, 5, 10, 15, 20, 25, and 30 µg/mL were made for ACV, and 1.25, 2.5, 5, 10, 15, 20, 25, 30, 40, and 50 µg/mL for VACV. The calibration curves were validated for absorbances recorded at 252 and 253 nm, respectively (UV-Vis spectrophotometer, Agilent 8453, Waldbronn, Germany).

### 2.6. Drug Loading and Release

ACV loading was tested, in triplicate, on the hydrogels NIP, NIP_200_, MIP_ACV_, MIP_A1:5_, MIP_A1:10_, and MIP_A1:15_. Each hydrogel disc (approx. 40 mg) previously washed and dried was placed in a tube with 5 or 15 mL of ACV aq. solution (0.3 mg/mL) and kept under oscillating agitation (300 rpm), at room temperature (23–25 °C), for 4 days. The absorbance of the solution was monitored at 252 nm (UV-Vis spectrophotometer, Agilent 8453, Waldbronn, Germany) by taking aliquots of 0.2 mL and diluting to 5 mL with ethanol:water mixture (50:50, *v*/*v*) (i.e., 1:25 dilution). The amount of drug loaded was estimated by the difference between the initial and final amount of drug in solution calculated using the previously prepared calibration curve, and referred to the unit of mass of the dry disc. 

VACV loading was evaluated, in triplicate, on the hydrogels NIP, NIP_200_, MIP_VACV_, MIP_V1:6_, MIP_V1:12_, and MIP_V1:32_. Each hydrogel disc (approx. 40 mg) previously washed and dried was placed in a tube with 5 mL of VACV (0.3 mg/mL) solution in 0.1 mM NaOH medium (pH 6.6). The loading tubes were kept under the same conditions of agitation, temperature, and time as for the ACV loading. The absorbance of the medium was monitored spectrophotometrically at 253 nm.

The drug network/water partition coefficient (*K_N/W_*) was calculated for each hydrogel from the total amount of drug loaded using the following equation

(2) $$Loading\;\left(total\right)=\frac{V_{S}+K_{N/W}*V_{p}}{W_{P}}*C_{0}$$

where *V_S_* is the volume of water absorbed by the hydrogel (mL), *V_p_* the volume of dry polymer (mL), *W_p_* the weight of the dry hydrogel (g), and *C*_0_ the concentration of drug in the loading solution (g/mL).

The loaded discs were removed from the tubes and rinsed with water. The surface water was removed with filter paper, and the discs were then immediately placed in release tubes with 15 and 10 mL of SLF (for ACV and VACV, respectively) under oscillating agitation (300 rpm) and at 35 °C. The release kinetics was evaluated for 24 h. Samples of the medium were periodically taken and analyzed following the same protocol as in Section 2.5.

Feasibility of the simultaneous loading and sterilization of the hydrogels was also investigated. Dried NIP, NIP_200_, MIP_VACV_, MIP_V1:6_, MIP_V1:12_, and MIP_V1:32_ hydrogels were placed in vials containing 5 mL of VACV (0.3 mg/mL) solution in 0.1 mM NaOH medium (pH 6.6) and kept for 12 h at room temperature. Then, the vials were steam heat sterilized (autoclave, 121 °C, 30 min) and stored at room temperature for 24 h without shaking. VACV solutions without hydrogels were processed as controls. Drug loading and release profiles were recorded as explained above. The sterilization tests were carried out in triplicate for each hydrogel and VACV solution and repeated in two independent runs.

### 2.7. Solvent Uptake

The uptake of water and SLF was monitored recording the increase in weight of dried discs after being immersed in 4 mL of the corresponding medium at room temperature (23–25 °C). At predetermined times (0.5, 1, 2, 4, 8, and 24 h), each disc was taken from the vial, excess water was removed with blotting paper, and the weight recorded. The discs were immediately returned to the vials. The solvent uptake was calculated as follows:

(3) $$Solvent\;uptake\;\left(\%\right)=\frac{W_{t}-W_{0}}{W_{0}}*100$$

where *W*_0_ and *W_t_* represent the weight of the dried and swollen hydrogel, respectively.

### 2.8. Light Transmission

The light transmittance (%) of discs swollen in SLF was measured in a spectrophotometer (Agilent Cary 60 UV-Vis, Waldbronn, Germany) in triplicate from 200 to 800 nm.

### 2.9. Mechanical Properties

The mechanical properties of NIP, NIP_200_, MIP_VACV_, MIP_V1:6_, MIP_V1:12_, and MIP_V1:32_ hydrogels swollen in water were tested in triplicate at room temperature (23–25 °C). Each hydrogel was cut into 16 × 9 mm strips and attached to the upper and lower clamps, with a 7 mm gap, on a TA.XT Plus Texture Analyzer (Stable Micro Systems Ltd., Surrey, UK), equipped with a 5 kg load cell. The crosshead speed applied to record the stress–strain plots was 0.1 mm/s. The Young’s modulus (*E*) was calculated as the slope of the straight-line part of engineering stress (force per cross-sectional area, N/mm^2^) versus the engineering strain (change in active length divided by original length, mm/mm) [41,42] as follows:

(4) E=FA0ΔLL0

### 2.10. HET-CAM Test

The hen’s egg test on chorioallantoic membrane (HET-CAM) was performed by incubating fertilized hen eggs (50–60 g) at 37 °C and 60% RH for 9 days. On the ninth day of incubation, a circular cut was made on the top of the egg of approximately 1 cm diameter with a rotary saw (Dremel 300, Breda, The Netherlands). The shell was removed, and the inner membrane was moistened with 0.9% NaCl for 30 min (time during which the egg remained inside the climatic chamber). The membrane was then removed to expose the chorioallantoic membrane (CAM) [33]. The test was performed by placing in each CAM a hydrogel disc previously soaked for 4 days in drug loading solution. Aqueous solutions of NaOH 0.1 N and NaCl 0.9% (300 µL) were used as negative and positive controls, respectively. The blood vessels were observed under white light for 5 min, to detect possible bleeding, vascular lysis, or coagulation. All tests were performed in triplicate and the irritation score calculated as reported previously [33].

### 2.11. Cornea and Sclera Permeability Tests

Fresh bovine and porcine eyes were collected from a local slaughterhouse and transported according to the Bovine Corneal Opacity/Permeability (BCOP) test protocol [33,43]. During transport, the eyes were kept immersed in PBS with added antibiotics (penicillin 100 IU/mL and streptomycin 100 μg/mL), in an ice bath. Corneas and scleras were isolated using a scalpel. The tissues were washed with 0.9% NaCl and mounted in vertical diffusion cells (Franz cells). To balance the tissues, the donor and receptor chambers were filled with carbonate buffer pH 7.2 and placed in a bath at 37 °C, with magnetic stirring, for 30 min. After that time, the content of the donor chamber was removed and the corneas and scleras were exposed to VACV-loaded NIP and MIP_V1:12_ discs (loaded as in Section 2.6). The discs were covered with 2 mL of 0.9% NaCl. In parallel, corneas and scleras were exposed to 2 mL of a VACV solution (100 µg/mL in NaOH 0.1 mM; pH 6.6) as a control, for 6 h. The donor chambers were covered with parafilm to avoid evaporation. Samples (1 mL) of the receptor medium were taken at 0.5, 1, 2, 3, 4, 5, and 6 h, and replaced with carbonate buffer pH 7.2 taking care of preventing bubbles formation in the diffusion cell. 

The amount of VACV permeated into the receptor chamber was quantified by HPLC (Autosampler Waters 717, Waters Controller 600, Photodiode Detector 996, Milford, MA, USA), equipped with a C18 column (Waters Symmetry C18, 5 μm, 4.6 × 250 mm) and operated with the Empower2 software. The mobile phase consisted of acetic acid (1:1000): methanol (90:10 *v*/*v*) with a flow rate of 1 mL/min. The injection volume was 50 µL and the column was kept at 30 °C. The calibration was performed with standard solutions of ACV and VACV (6.25–0.19 µg/mL) in carbonate buffer pH 7.2 [44], and the absorbance was quantified at 251 nm. Retention times were ~3 min for VACV and 4.8 min for ACV (typical HPLC chromatograms are shown in Figure S1, Supporting Information). The accumulated amounts of drug permeated were calculated from the sum of VACV and ACV peaks [20]. The steady-state flow (*J*) and the time delay (t_lag_) were obtained from the slope and x-intercept, respectively, of the linear regression of the accumulated amount of drug permeated per area vs. time [45]. After 6 h of the test, aliquots of the liquid remaining at the donor chamber were taken for further analysis. The coefficients of permeability of the drug through the cornea and sclera were calculated as the ratio of *J* to the concentration of drug in the donor chamber [45].

The corneas/scleras were also removed from the diffusion cells after 6 h test, rinsed with 0.9% NaCl, and immersed in 3 mL of an ethanol:water mixture (50:50 *v*/*v*) overnight. They were then sonicated for 99 min at 37 °C, centrifuged (1000 rpm, 5 min, 25 °C), filtered, and re-centrifuged (14,000 rpm, 20 min, 25 °C) [46]. The drug extracted from the corneas/scleras was quantified by HPLC as explained above.

### 2.12. Statistical Analysis

The effects of hydrogel composition on drug loading and permeability through porcine and bovine tissues were analyzed using ANOVA and the multiple range test (Statgraphics Centurion XVII, Stat Point Technologies Inc., Warrenton, VA, USA).

## 3. Results and Discussion

### 3.1. Computational Modeling

**Figure 2:**
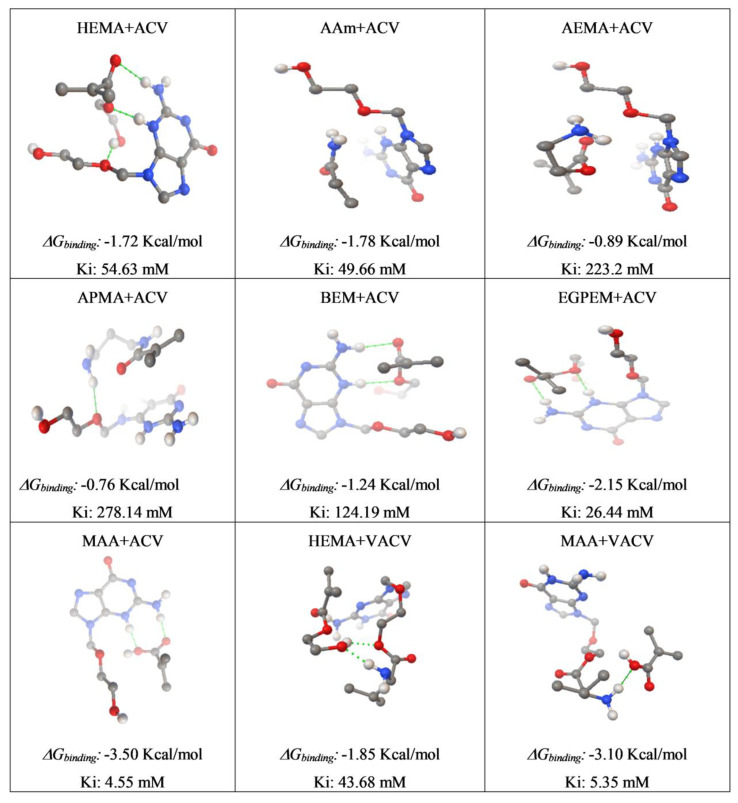
Computational modeling results of the interaction of acyclovir (ACV) with the monomers 2-hydroxyethyl methacrylate (HEMA), acrylamide (AAm), 2-aminoethyl methacrylate hydrochloride (AEMA), N-(3-aminopropyl) methacrylamide hydrochloride (APMA), butoxyethyl methacrylate (BEM), ethylene glycol phenyl ether methacrylate (EGPEM), and methacrylic acid (MAA); and the interaction of valacyclovir (VACV) with 2-hydroxyethyl methacrylate (HEMA) and methacrylic acid (MAA).

Computational modeling is a versatile tool for the first screening of functional monomers suitable for preparing imprinted hydrogels [34,47,48]. Although the conditions during polymerization cannot be precisely resembled, computational modeling has been shown to be useful to identify the monomers with a high affinity for the template drug, which, in turn, may endow the imprinted hydrogels with high rebinding and controlled release performances [48,49]. HEMA, as a main structural monomer of SCLs, and six functional monomers bearing different chemical moieties (amido, amine, phenyl, butyl, and acrylic acid) were screened regarding their interactions with ACV and VACV. Results of computational modeling are summarized in Figure 2. 

High negative values of *ΔG_binding_* indicated favorable binding interactions between the drug and the monomer. The lower the value of *Ki*, the lower the likelihood of complex disassembly. Considering both *ΔG_binding_* and *Ki*, the interaction of ACV and VACV with MAA was predicted to be more favorable than with other monomers (Figure 2). Interestingly, MAA may interact with VACV through hydrogen bonds with the amino groups in the aromatic ring (as in the case of ACV) and also with the primary amine of the valine side chain. The pKa of the amino group of valine has been estimated to be in the 9.1–9.6 range [50]. Thus, in the aqueous medium used to load the hydrogels and in the lacrimal fluid, this amino group is expected to remain protonated. This opens the possibility of the fact that the MAA mers in the hydrogels may readily interact with VACV through electrostatic interactions. Therefore, MAA was chosen as the functional monomer to prepare the hydrogels.

### 3.2. Synthesis of Hydrogels and Drug Removal

Imprinted and non-imprinted hydrogels were synthesized combining HEMA with MAA as the functional monomer. The drug was added at different levels, in ascending mole ratios of ACV:MAA (1:5, 1:10, and 1:15 mol/mol) and VACV:MAA (1:6, 1:12, and 1:32 mol/mol), as explained in Table 1. It should be noted that the total content in MAA was fixed, and only the content in the template drug varied. The ratio VACV:MAA was lower than that of ACV:MAA because of the additional binding points that VACV may establish with MAA according to the computational study. The use of MAA as comonomer should enhance drug–hydrogel interactions through hydrogen bonding of the acrylic acid group with the rings of the drugs (ACV and VACV) and electrostatic interactions with the VACV side chain. The highest drug:MAA mole ratio was limited by the poor solubility of the drugs in the monomers solution, but also considering that 1:4 to 1:6 mole ratios have been commonly reported as adequate to create imprinted cavities [34,51]. Additionally, non-imprinted hydrogels prepared with the same content in MAA (NIP_200_) and imprinted hydrogels without MAA (MIP_ACV_ and MIP_VACV_) were synthesized under the same conditions in order to elucidate the role of the functional monomer and of the drug template, respectively.

**Figure 3:**
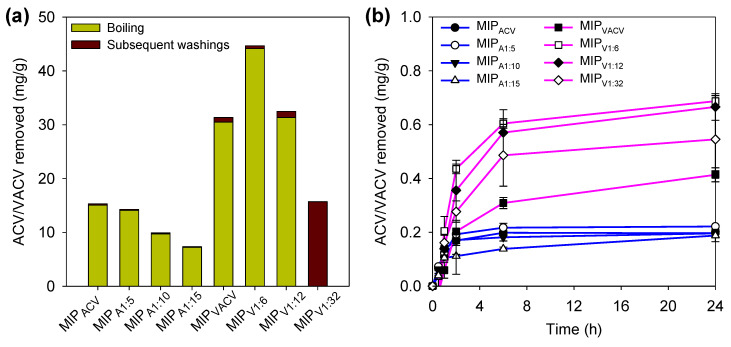
(**a**) Amounts of ACV and VACV removed during washing in boiling water and subsequent washings from each of the hydrogels tested (codes as in Table 1), and (**b**) release profiles of ACV and VACV from the imprinted discs that were previously boiled in water (15 min) and dried to constant weight. The drug released corresponds to that used as a template during synthesis.

Monomers and template drug molecules were easily detected in the washing medium. Monomers mainly absorbed at wavelengths below 220 nm, while ACV and VACV absorbed at 252–253 nm. Both peaks were clearly recorded in the first washing solution after boiling. VACV was easily removed from the VACV-imprinted hydrogels during the washing process in boiling water (Figure 3a). Elution of residual monomers caused minor interferences in the quantification of the amounts of drug extracted. By contrast, the amounts of ACV removed from the hydrogels synthesized using ACV as a template were lower than the amounts added during synthesis (Figure 3a). This could be due to the limited solubility of the ACV in aqueous medium, which may favor hydrophobic association with the hydrogel polymer backbone.

### 3.3. Direct Drug Release Test from Boiled Hydrogels

A direct release in SLF was carried out for hydrogels after boiling (without further washing) for the first screening of their ability to release the remaining ACV and VACV template in a sustained way (Figure 3b). The hydrogels polymerized using ACV as a template (MIP_ACV_, MIP_A1:5_, MIP_A1:10_, MIP_A1:15_) released about 0.20 mg of drug per gram of disc. The sum of the amount released plus the amount already removed during boiling was still lower than the total amount added during synthesis (45, 45, 23, and 15 mg of ACV, respectively), and practically no differences were observed between the different types of hydrogel. This finding pointed to ACV template entrapment in the polymer network, favored by the hydrophobicity of the drug. By contrast, the hydrogels polymerized using VACV as the template (MIP_VACV_, MIP_V1:6_, MIP_V1:12_, MIP_V1:32_) released the small amount of remnant VACV in a sustained way for 24 h. The mass balance of VACV confirmed that the molecules used as the template were mostly removed during the boiling process. A similar release experiment was performed with non-imprinting hydrogels and, as expected, no signal was recorded at the wavelength used for drug quantification.

### 3.4. Drug Loading

**Figure 4:**
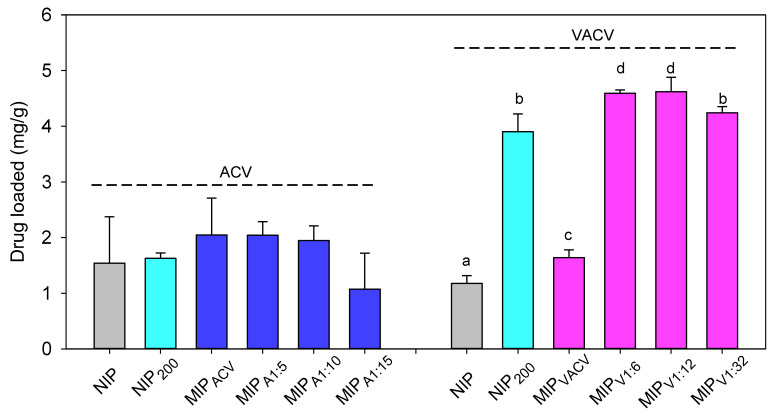
Amounts of ACV and VACV loaded by the hydrogels after 4 days of soaking in the drug solution (0.3 mg/mL). Different letters indicate statistically significant differences with a *p*-value < 0.05. On the X-axis, the hydrogel codes are the same as those in Table 1.

The loading of both ACV and VACV was carried out in aqueous medium in the absence of organic co-solvents and salts to avoid interferences in the binding to the network. Only in the case of VACV, the solution was prepared in diluted NaOH medium (0.1 mM) to increase the pH from 4.0 to 6.6, a more biocompatible value. ACV-imprinted hydrogels showed low capability to reload the drug (Figure 4). The presence of MAA did not significantly favor the subsequent loading capacity of the hydrogel. In addition, an increase in the ACV loading volume from 5 to 15 mL did not cause any improvement. By contrast, MAA notably enhanced the loading of VACV, and a clear difference (*p* < 0.05) was observed between imprinted (MIP_V1:6_ and MIP_V1:12_) and non-imprinted (NIP_200_) hydrogels (Figure 4). The MAA functionalization significantly improved the total amount of VACV loaded by the discs after 4 days of soaking in the drug solution. The higher affinity of the hydrogels for VACV can be explained by the stronger interaction of MAA with the amino group of the lateral chain.

The network/water partition coefficient (*K_N/W_*) of VACV was calculated for both imprinted and non-imprinted hydrogels. Control hydrogels, i.e., NIP without MAA (NIP), had *K_N/W_* values of 3.0 (s.d. 0.4). The value obtained for the hydrogels imprinted without MAA (MIP_VACV_) was 4.4 (s.d. 0.4). *K_N/W_* values higher than 1 indicated that the drug was hosted both in the water phase and interacting with the polymer network [26,33,52]. Hydrogels containing MAA mers showed remarkably higher *K_N/W_* values and ranked as follows: NIP_200_ (11.3; s.d. 1.0) = MIP_V1:32_ (12.3; s.d. 0.3) < MIP_V1:6_ (13.4; s.d. 0.1) = MIP_V1:12_ (13.5; s.d. 0.8). Interestingly, simple addition of VACV to the HEMA monomers (MIP_VACV_ hydrogels imprinted without MAA) slightly increased the amount of drug loaded. This finding suggests that the presence of VACV molecules and their interaction with HEMA (although weak according to the computational modeling) may have created hosting cavities and channels in the network that facilitated subsequent loading. Nevertheless, this effect was much lower than that achieved when truly imprinted networks were prepared. MIP_V1:6_ and MIP_V1:12_ showed the highest loading capability. MIP_V1:32_ was less efficient as there was an excess of MAA mers per drug molecule, which means that most MAA groups are randomly distributed along the hydrogel network as in the case of NIP_200_. A similar phenomenon has previously been reported for other imprinted hydrogels [51].

### 3.5. Drug Release

**Figure 5:**
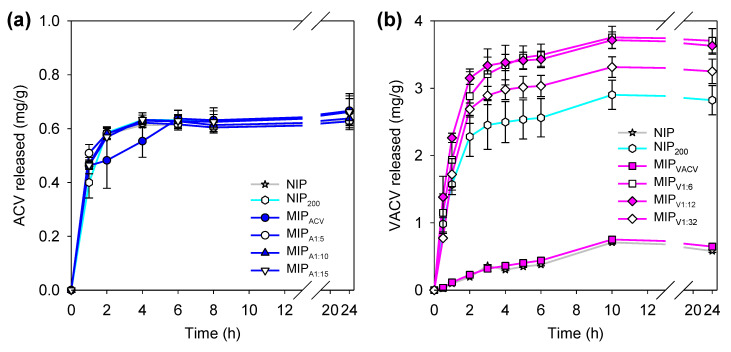
Release profiles of (**a**) ACV and (**b**) VACV from non-imprinted and imprinted hydrogels in simulated lacrimal fluid (SLF).

Drug-loaded discs (as referred above) were immersed in SLF under sink conditions to evaluate the capability of the hydrogels to control the release. For the discs loaded with ACV, a plateau was reached after approximately 6 h of testing, with a maximum amount released of 0.6 mg/g of disc (Figure 5a). Again, the discs loaded with ACV were only capable of releasing half the amount of drug loaded in spite of the fact that the test was carried out under sink conditions. The release profiles were similar to each other. No significant differences in the amounts of ACV released were recorded for the different types of hydrogel. Thus, hydrogels loaded with ACV were discarded for subsequent tests because of the irreversible binding of a relevant portion of the drug, which may compromise attaining the minimum level required for antiviral activity (IC_50_: 1.92 µg/mL) [53].

By contrast, the discs loaded with VACV showed a sustained release profile for 10 h (Figure 5b). In general, the hydrogels were able to release most of the amount of drug loaded. Specifically, hydrogels functionalized with MAA and imprinted with VACV released higher amounts than their homologous counterparts without MAA (MIP_VACV_) (Figure 5b). No significant differences were found between MIP_V1:12_ and MIP_V1:6_, in good agreement with their similar loading capabilities (Figure 4). The combination of MAA functionalization and molecular imprinting clearly improved the capacity of the hydrogels to host the drug and to release it in a sustained way.

### 3.6. Hydrogels Characterization

**Figure 6:**
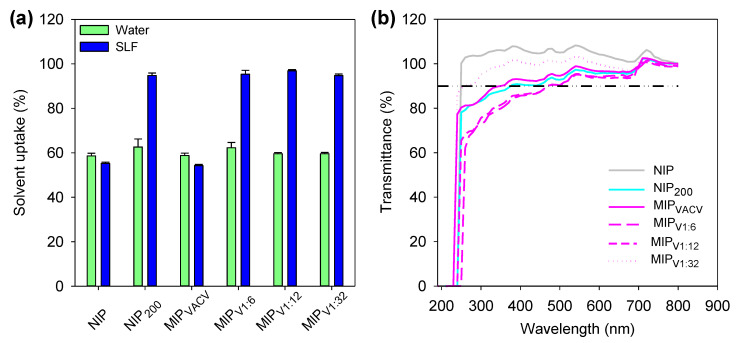
(**a**) Solvent (water or SLF) uptake values recorded for the hydrogels after 24 h of soaking (hydrogels codes as in Table 1), and (**b**) light transmittance values of hydrogels swollen in SLF. The acceptance value of 90% transmittance is shown as a dash-dot line.

Hydrogels NIP, NIP_200_, MIP_VACV_, MIP_V1:6_, MIP_V1:12_, and MIP_V1:32_ were characterized in terms of solvent uptake, and transmittance and mechanical properties. When immersed in water or SLF, all hydrogels sorbed the medium rapidly, reaching the equilibrium within one hour. The solvent uptake in water reached values close to 60% (Figure 6a). Similar values were recorded for hydrogels without MAA when immersed in SLF. By contrast, HEMA-MAA networks (both non-imprinted and imprinted) were able to absorb up to 90% due to the ionization of MAA in SLF (pKa = 4.7) [54,55]. A similar swelling was also observed in the VACV loading solutions (pH 6.6). The water uptake values were in the typical range of hydrophilic contact lenses [56]. In addition, all hydrogels showed excellent light transmission properties (Figure 6b), with transmittance values above 90% at 600 nm.

Concerning the mechanical properties, the tensile strength tests showed that all types of hydrogels had a Young’s modulus close to 0.50 MPa (Table 2). The values were within the typical range of hydrophilic contact lenses, so they would be clinically valid [42]. The similiarities in solvent uptake and mechanical properties observed between imprinted and non-imprinted hydrogels indicated that the presence of VACV had a minor impact on the polymerization process. 

### 3.7. Sterilization Tests

With a view to scale-up and clinical use, feasibility of steam heat sterilization and the loading of the hydrogels in one single step was tested. Although control VACV solutions had similar absorbance values at 253 nm before and after sterilization recorded using UV spectrophotometry, HPLC analysis revealed the hydrolysis of this prodrug in the parent drug (Figure S2 in Supplementary Material). The quantification of the areas indicated that 30% of VACV transformed into ACV after steam heat sterilization (the values were consistently reproducible in two independent runs). This stability problem agreed with a previous report [57]. Although the pH was kept closer to neutrality (6.6) compared to previous studies [57], it seems that heating at 121 °C may have accelerated the hydrolysis process. Moreover, a significant decrease in the pH of the VACV loading solutions occurred during sterilization both in the absence and presence of the hydrogels, to values in the 4.0–4.2 range. The decrease in the pH to values below the pKa of MAA (4.7) [54,55] caused the acrylic acid groups in the hydrogels to be less ionized, which was evidenced by a decrease in the swelling degree compared to that recorded in SLF (Figure 6a) or in the non-sterilized VACV loading solutions. Sterilized hydrogels showed the same solvent uptake (~60%) disregarding their content in MAA. 

The decrease in the VACV content together with the lower ionization of MAA (i.e., decreased likelihood of ionic interactions) and swelling degree (i.e., lower mesh size may hinder drug diffusion) of the hydrogels explained the remarkably lower drug loading recorded for the hydrogels after sterilization: 0.35–0.37 mg/g for NIP and MIP_VACV_, and 0.75–0.80 mg/g recorded for NIP_200_, MIP_V1:6_, MIP_V1:12_, and MIP_V1:32_. Drug release profiles (Figure S3 in Supplementary Material) revealed the complete release of the drug loaded in the first two hours of immersion in SLF. This rapid release suggests that VACV was loaded mainly by interacting with the outer layers of the hydrogels. Therefore, the scale-up of VACV-loaded hydrogels may require the steam heat sterilization of the hydrogels first and then the conditioning in VACV solution prepared using sterilizing filtration. For the subsequent tests, the hydrogels were handled under clean conditions, and VACV loading was carried out as reported in Section 3.4.

### 3.8. HET-CAM Test

**Figure 7:**
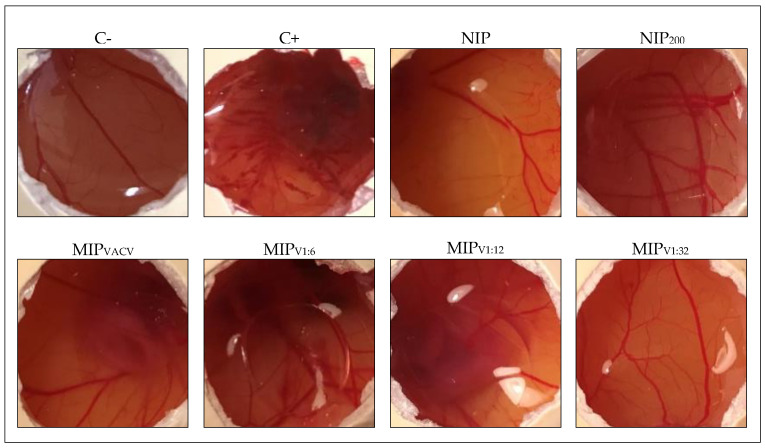
Pictures of chorioallantoic membranes during the hen’s egg test on chorioallantoic membrane (HET-CAM) test after 5 min of contact with VACV-loaded hydrogels. The negative (C-) and positive (C+) controls refer to 0.9% NaCl and 0.1 N NaOH solutions, respectively.

The HET-CAM test was used for a first screening of ocular tolerance of the developed hydrogels. The chorioallantoic membrane resembles the vasculature of conjunctiva and its responsiveness against irritant substances [58]. The hydrogels were first loaded with VACV and then placed on the chorioallantoic membrane. No hemorrhage, lysis, or coagulation was observed for any of the six types of hydrogel tested, behaving in the same way as the negative control (NaCl 0.9%) with an irritation score of 0 (Figure 7). This finding is in good agreement with the excellent safety profile of VACV when the retina was exposed to concentrated solutions of this drug [15].

### 3.9. Corneal and Scleral Permeability Test

Permeability tests were carried out with the hydrogels showing more promising results in the former assays, namely MIP_V1:12_. A VACV solution (100 µg/mL) containing the same amount of drug as the MIP_V1:12_ hydrogel was used as a reference. VACV-loaded NIP hydrogels were also used as a control. The tests were carried out using cornea and sclera from both the bovine and porcine source. Bovine tissues are recommended as an alternative to in vivo animal tests of ocular tolerance and permeability to new substances [59]. Nevertheless, porcine eye tissues resemble better the structure of human ones in terms of composition and thickness [60]. Therefore, the findings of the study may be more robust using both tissue sources.

**Figure 8:**
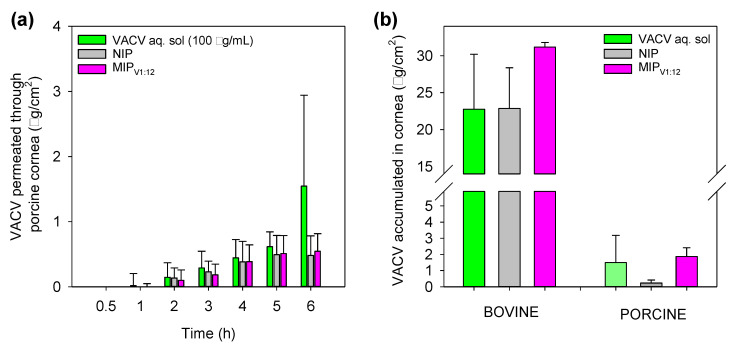
Amounts of VACV permeated through porcine cornea (**a**) and accumulated in bovine or porcine cornea (**b**) when applied as a drug solution or as VACV-loaded NIP and MIP_V1:12_ hydrogels. Mean values (*n* = 3); error bars indicate standard deviations.

At the corneal level, VACV permeated very slowly through the porcine cornea, and only after one hour in contact with the drug solution, quantifiable levels were recorded (Figure 8a). The lag time for the drug released from the hydrogels was two hours. In the case of the bovine cornea, the VACV detected in the receptor compartment was below the limit of quantification. Nevertheless, the accumulation of VACV in the bovine cornea was remarkable (Figure 8b), being well above 20 µg/cm^2^ for the three formulations. Accumulation was also observed in the porcine cornea although at lower levels, which may, in part, be related to its lower thickness [61]. Drug accumulation in the porcine cornea showed a clear dependence on the concentration gradient that the formulation provided on the ocular surface; namely, MIP_V1:12_ and VACV solution favored the highest accumulations. These findings suggest high affinity for cornea tissue probably due to the presence of specific transporters in the epithelium, although the changing polarity in the cornea layers may be responsible for the lack of progression toward the receptor mimicking the aqueous humor. Thus far, the highest accumulation of VACV into the cornea has been reported for VACV-loaded lipid nanocarriers formulated as eye drops applied to goats that led to 30 µg drug per cm^2^ of cornea [19]. Similar VACV accumulation values were provided by MIP_V1:12_ hydrogels in bovine corneas (Figure 8b).

**Figure 9:**
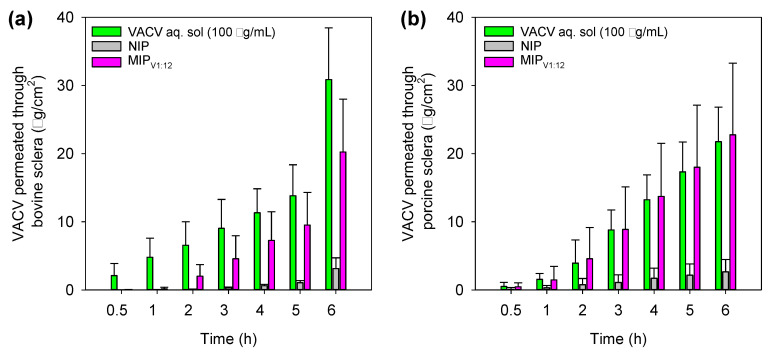
Amounts of VACV permeated through (**a**) bovine sclera and (**b**) porcine sclera when applied as a drug solution or as VACV-loaded NIP and MIP_V1:12_ hydrogels. Mean values (*n* = 3); error bars indicate standard deviations.

Regarding the sclera tests, VACV permeated more easily and could be quantified from the first sampling at 0.5 h (Figure 9). The hydrophilicity and small size of VACV explain the fast pass through the sclera [57]. As expected, VACV permeation was initially faster when applied as a solution. Nevertheless, as the MIP_V1:12_ hydrogels may release most drugs in the scale frame of the test (see Figure 5), the amounts of VACV released from the hydrogels could cross the sclera as efficiently as in the case of the solution.

It should be taken into account that the data recorded for the VACV solution imply that the tissue is exposed to a high drug concentration from the first second of the study and that the eye drop remains on the tissue for several hours, which is not feasible in vivo. By contrast, the hydrogel may be used for daily wearing and delivering the drug at a rate that allows efficient penetration in the ocular tissues; thus, better in vivo performance can be expected. For comparison purposes, the steady-state flow (*J*) was estimated in the 1–5 h of the test for all formulations and for both animal species (Table 3). MIP_V1:12_ hydrogels provided drug flux levels as large as the VACV solution, and one order of magnitude larger than control NIP hydrogels (statistically significant differences, ANOVA, *p* < 0.05). VACV did not significantly accumulate in the sclera, but passed through it. Higher flux values recorded for porcine sclera can be related to their smaller thickness (73.2 ± 2.7 μm) compared to that of bovine sclera (129.8 ± 14.7 μm) as experimentally measured [61]. The permeation coefficient (*P*_app_) was estimated referring the flux to the drug concentration measured in the donor chamber at the end of the test. No statistically significant differences (ANOVA, *p* = 0.6832) were observed among the formulations in either bovine or porcine scleras (Table 3). This means that VACV can permeate through the sclera once released from the hydrogels as the free drug in solution does. Therefore, imprinted hydrogels loaded with VACV may act as drug-release platforms with the advantage over aqueous solutions that the CLs can provide sustained release, minimizing drugs lost and non-productive absorption. Although the design of drug-imprinted networks involves additional steps (polymerization in the presence of the drug, drug removal, drug reloading) compared to the direct loading of preformed non-imprinted networks, the obtained results of drug loading and tissue permeation evidence the value of the molecular imprinting approach in the case of VACV.

## 4. Conclusions

Acyclovir and valacyclovir behaved differently when incorporated into HEMA-based hydrogels, despite their similar chemical structure and a priori similar binding energy with MAA. The limited solubility of ACV in the monomer mixture together with unspecific hydrophobic interactions may explain why ACV-imprinted hydrogels were not effective in terms of drug loading and release. Moreover, the small amount of ACV loaded was not completely released in SLF, probably because of the hydrophobicity of the drug. By contrast, the use of MAA as functional monomer remarkably increased the affinity of the hydrogels for VACV through electrostatic interactions between the acrylic acid group of the monomer and the drug lateral chain. The use of VACV as a template during polymerization facilitated the arrangement of the polymer network, creating specific cavities that contributed to enhancing the affinity of the drug for the hydrogel in subsequent loading. The degree of swelling, light transmission, and mechanical properties showed common values to daily wear contact lenses. In addition, no potential eye irritation was observed in the HET-CAM assay. VACV-imprinted hydrogels can release the drug in a sustained manner for 10 h, which is a common time of wearing disposable SCLs. Therapeutically relevant amounts accumulated in the cornea. At the sclera level, VACV-loaded hydrogels showed permeability values equivalent to those achieved with the aqueous solution of the drug. VACV permeability through the sclera suggests the possibility of delivery to the posterior segment. Therefore, hydrogels containing MAA and imprinted with VACV are suitable candidates for the preparation of drug-eluting contact lenses. As VACV does not withstand steam heat sterilization, the scale-up may involve the autoclaving of the hydrogels first and then the packaging in VACV solution prepared using sterilizing filtration.

## Figures and Tables

**Table 1:** Composition of the hydrogels (NIP: Non-imprinted hydrogels, MIP: Imprinted hydrogels). Final ethylene glycol dimethacrylate (EGDMA), methacrylic acid (MAA), and 2,2′-azo-bis(isobutyronitrile) (AIBN) concentrations were 8, 200 and 10 mM, respectively.

Hydrogel	HEMA (mL)	EGDMA (µL)	MAA (mL)	ACV (mg)	VACV (mg)	AIBN (mg)
**NIP**	5	7.55	0	0	0	8.21
**NIP_200_**	5	7.55	0.084	0	0	8.21
**MIP_ACV_**	5	7.55	0	45	0	8.21
**MIP_A1:5_**	5	7.55	0.084	45	0	8.21
**MIP_A1:10_**	5	7.55	0.084	23	0	8.21
**MIP_A1:15_**	5	7.55	0.084	15	0	8.21
**MIP_VACV_**	5	7.55	0	0	25	8.21
**MIP_V1:6_**	5	7.55	0.084	0	50	8.21
**MIP_V1:12_**	5	7.55	0.084	0	25	8.21
**MIP_V1:32_**	5	7.55	0.084	0	10	8.21

**Table 2:** Elastic properties of the hydrogels.

Hydrogel	Young’s Modulus (MPa)
NIP	0.547
NIP_200_	0.358
MIP_VACV_	0.536
MIP_V1:6_	0.623
MIP_V1:12_	0.538
MIP_V1:32_	0.548

**Table 3:** Flux (*J*), permeability coefficient (*P*_app_), and drug accumulated in bovine and porcine sclera when VACV was applied as a solution or as VACV-loaded NIP and MIP_V1:12_ hydrogels. Mean values (*n* = 3) and error bars indicate standard deviations; n.d. means non-quantifiable values.

Formulation	Bovine Sclera			Porcine Sclera		
	*J* (μg/(cm^2^·h))	*P*_app_ (× 10^6^) (cm/s)	VACV Accumulated (μg/cm^2^)	*J* (μg/(cm^2^·h))	*P*_app_ (× 10^6^) (cm/s)	VACV Accumulated (μg/cm^2^)
VACV sol.	2.28 (0.35)	8.79 (1.37)	0.58 (0.41)	4.09 (0.72)	13.82 (2.46)	1.13 (0.51)
NIP	0.27 (0.06)	8.78 (2.13)	n.d.	0.42 (0.32)	9.75 (7.70)	0.12 (0.13)
MIP_V1:12_	2.40 (1.15)	12.91 (6.23)	0.17 (0.02)	4.23 (1.74)	11.37 (4.67)	1.40 (0.42)

## Supplementary Materials

The following are available online at https://www.mdpi.com/2073-4360/12/9/2026/s1, Figure S1: Typical HPLC chromatograms of VACV and ACV standard solutions and of a sample of the receptor medium during the permeability tests. Figure S2. HPLC chromatograms of the VACV loading solution before and after steam heat sterilization (autoclave 121 °C, 30 min). Figure S3. Drug (VACV+ACV) release profiles in SLF from non-imprinted and imprinted hydrogels that were loaded by soaking in VACV solution and sterilized by steam heat sterilization. The data are shown as accumulated amounts obtained after conversion from absorbance values recorded at 253 nm.
