# Supplementary material for: Imprinted Contact Lenses for Ocular Administration of Antiviral Drugs
Source: Polymers (Basel). 2020 Sep 4;12(9):2026. doi: 10.3390/polym12092026 (PMC7565425; doi:10.3390/polym12092026)
Supplement: Supplementary file 1 [file polymers-12-02026-s001.pdf]

SUPPORTING INFORMATION

Imprinted contact lenses for ocular administration of antiviral drugs

Angela Varela-Garcia, José Luis Gomez-Amoza, Angel Concheiro and Carmen Alvarez-Lorenzo *

Departamento de Farmacología, Farmacia y Tecnología Farmacéutica, I+D Farma Group, Facultad de Farmacia and Health Research Institute of Santiago de Compostela (IDIS), Universidade de Santiago de Compostela, 15782 Santiago de Compostela, Spain; angela.varela.garcia@rai.usc.es; joseluis.gomez.amoza@usc.es; angel.concheiro@usc.es

* Correspondence: carmen.alvarez.lorenzo@usc.es; Tel.: +34 881815239 (C.A.L.)

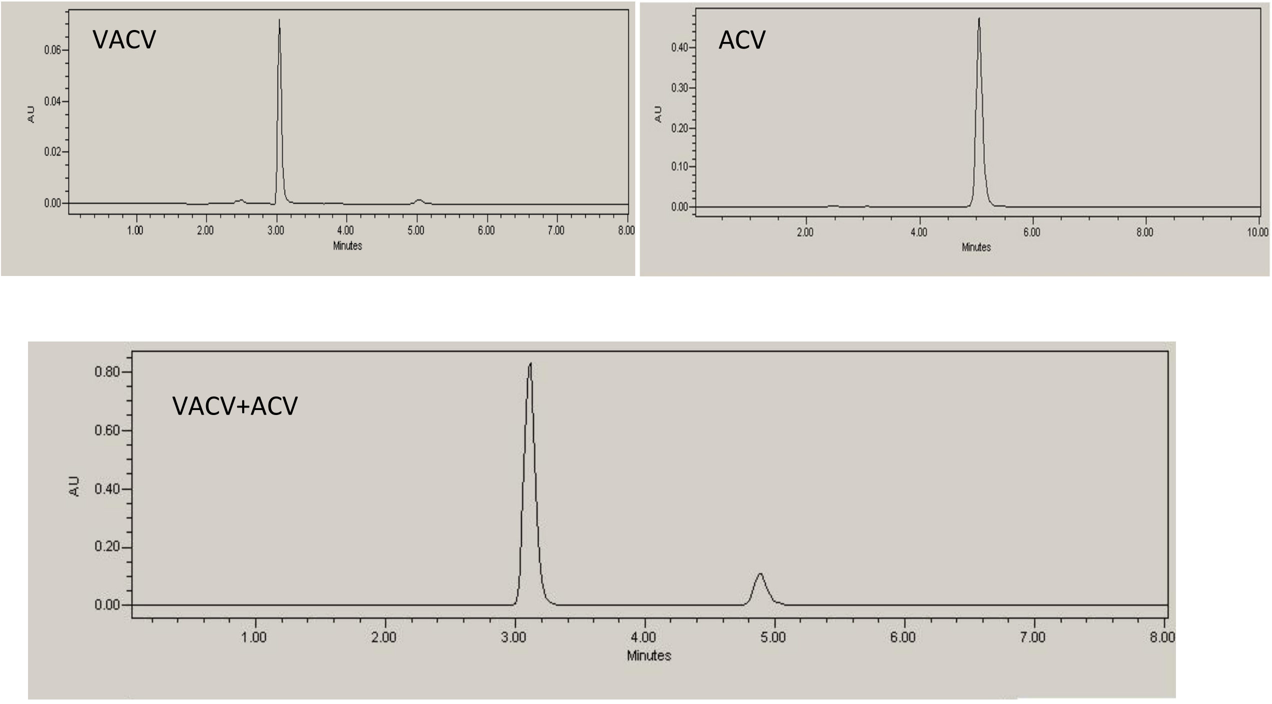

Figure S1. Typical HPLC chromatograms of VACV and ACV standard solutions (up) and of a sample of the receptor medium during the permeability tests (down).

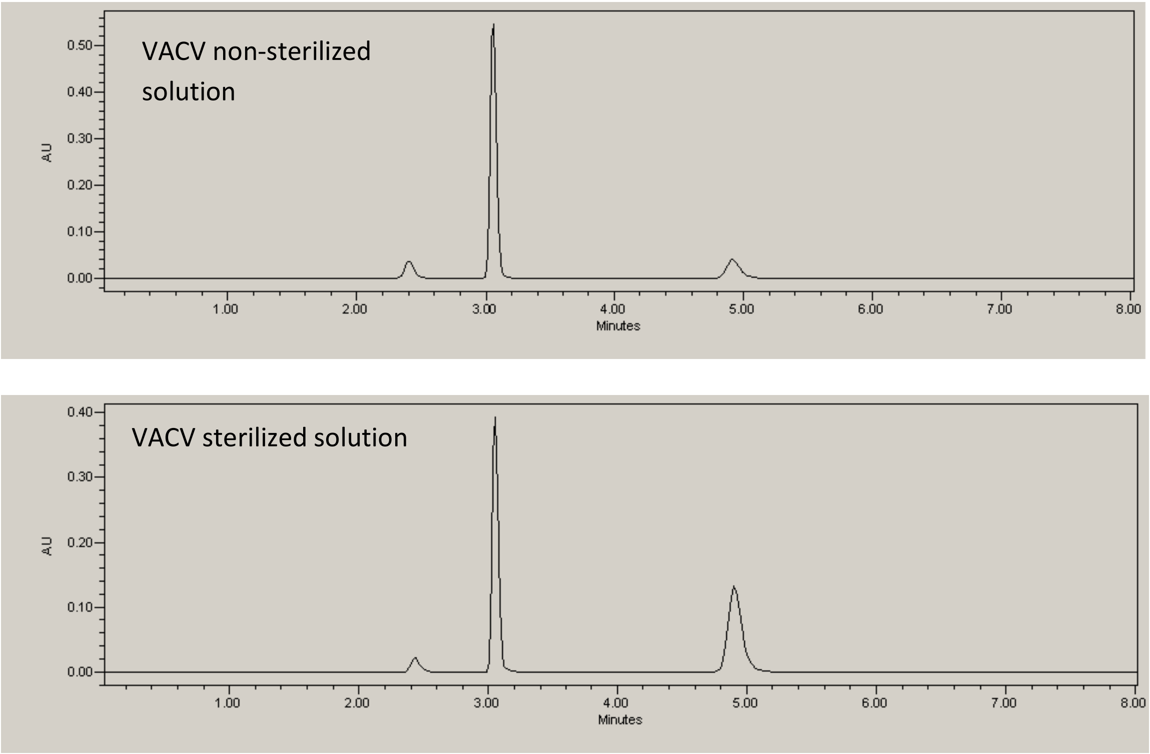

Figure S2. HPLC chromatograms of the VACV loading solution before (up) and after (down) steam heat sterilization (autoclave 121 ºC, 30 min).

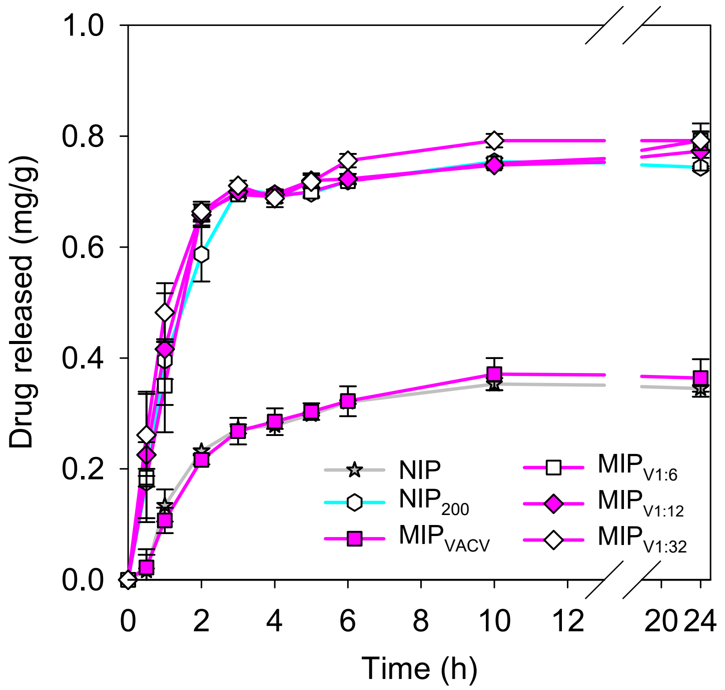

Figure S3. Drug (VACV+ACV) release profiles in SLF from non-imprinted and imprinted hydrogels that were loaded by soaking in VACV solution and sterilized by steam heat sterilization. The data are shown as accumulated amounts obtained after conversion from absorbance values recorded at 253 nm.
